# Prediction of outcome in severe traumatic brain injury: Vestibulo-ocular monitoring as a novel tool

**DOI:** 10.4103/2152-7806.74144

**Published:** 2010-12-22

**Authors:** Taopheeq Bamidele Rabiu

**Affiliations:** Department of Neurological Surgery, University College Hospital, Ibadan, Nigeria

Dear Sir,

Outcome prediction is a major part of the present-day management protocol for severe traumatic brain injury (sTBI). The introduction of vestibulo-ocular monitoring (VOM) using galvanic labyrinth polarization (GaLa) by Schlosser and his colleagues represents a novel method of assessing brainstem function and predicting outcome in sTBI. VOM is likely to replace oculo-cephalic and caloric tests in assessing brainstem function in the near future and may play an important role in the diagnosis of brain death.

Prediction of outcome in sTBI is an integral part of the present-day management of TBI. It allows proper stratification of patients and allocation of scarce resources in the overall planning of care. As discussed by Schlosser and his colleagues,[1] the use of prediction tools such as the post-resuscitation Glasgow Coma Score(GCS), electroencephalogram (EEG), electrical potentials (EPs), cranial computed tomography (CT) findings, cerebral metabolism measured by, for example, microdialysis and infrared spectroscopy, among others, is usually attended by several limitations. The authors’ introduction of VOM using GaLa represents a novel method of assessing brainstem function and predicting outcome in sTBI.

In their paper, the authors introduced VOM as a means of testing brainstem function. This is intended to complement the findings of brainstem imaging and thus should improve the prognostication of the long-term outcome in sTBI. They performed VOM through video-oculographic (VOG) recording of eye movements during GaLa stimulation of both labyrinths. The eye movement response is elicited via the vestibulo-ocular reflex arc (VOR), i.e. via the afferents from the peripheral neurons to the vestibular nuclei and, subsequently, to the oculomotor neurons.

They performed the test in comatose sTBI patients within 3 days of injury while on intensive care unit (ICU) admission and recorded the oculomotor response (OMR) to stimulation of the labyrinths. In their study of 26 patients, GaLa induced OMR in 15 (57.7%) but not in 11 (42.3%) patients. All the patients in the latter group had an unfavorable Glasgow outcome score (GOS) of 1–2, with death in 10 (90.9%) of them within 15 days of trauma despite operative interventions in six of them. VOM is likely to replace oculo-cephalic and caloric tests in assessing brainstem function in the near future and may play an important role in the diagnosis of brain death. Its depression by muscle relaxants is a considerable limitation of its usefulness as it may preclude its application in many sTBI patients who are often given such agents to allow for adequate ventilation and optimization of metabolism in the early phase of ICU admission. Thus, it may be worthwhile to perform VOM later than the first 3 days after TBI used by the authors to increase its global usage. This will no doubt require an evaluation of its correlation with the eventual outcome when performed during this period. The authors’ stratification of patients into favorable outcome (GOS > 2) and unfavorable outcome (GOS < 3) is at variance with the established convention, in which the five outcome categories are collapsed into a dichotomous distinction between, on one hand “poor” or “unfavorable” outcome (dead, vegetative state or severe disability) and, on the other, an “independent” or “favorable” outcome (moderate disability or good recovery).[2] Proper stratification would have given a *P*-value of 0.004 and not the <0.001 stated by the authors. This, however, does not appear to undermine the predictive value of OMR between the two groups.
